# Computed Tomography‐Guided Biopsy of a Ureteral Urothelial Carcinoma Mimicking a Submucosal Bladder Tumor at the Ureterovesical Junction

**DOI:** 10.1002/iju5.70132

**Published:** 2025-12-21

**Authors:** Akira Ohtsu, Yuji Fujizuka, Seiji Arai, Masakazu Yamaguchi, Hiroyuki Tokue, Tatsuro Maehara, Takanori Shimizu, Yoshitaka Sekine, Hayato Ikota, Kazuhiro Suzuki

**Affiliations:** ^1^ Department of Urology Gunma University Hospital Maebashi Gunma Japan; ^2^ Department of Diagnostic and Interventional Radiology Gunma University Hospital Maebashi Gunma Japan; ^3^ Clinical Department of Pathology Gunma University Hospital Maebashi Gunma Japan

**Keywords:** computed tomography‐guided biopsy, intramural ureteral cancer, submucosal bladder tumor, trans‐extraperitoneal and transvesical approach, urothelial carcinoma

## Abstract

**Introduction:**

Intramural ureteral urothelial carcinoma can be difficult to diagnose, especially when presenting without mucosal abnormalities or positive cytology. In such cases, percutaneous biopsy may provide an alternative diagnostic approach.

**Case Presentation:**

A 74‐year‐old man presented with acute renal failure caused by bilateral hydronephrosis. Cystoscopy showed no mucosal abnormality. Retrograde pyeloureterography revealed bilateral distal ureteral strictures, but urine cytology from ureteral catheters was negative. Imaging revealed a submucosal mass on the right dorsal bladder wall. Urothelial carcinoma was diagnosed on a computed tomography‐guided percutaneous trans‐extraperitoneal and transvesical biopsy. Neoadjuvant chemotherapy was administered and radical cystectomy performed. Pathology confirmed invasive urothelial carcinoma originating from the right intramural ureter at the ureterovesical junction with bladder muscle invasion.

**Conclusion:**

This case highlights the diagnostic challenges of intramural ureteral urothelial carcinoma presenting as a submucosal bladder tumor. Computed tomography‐guided percutaneous biopsy can be a safe and effective diagnostic option in such challenging cases.

## Keynote Message

Intramural ureteral urothelial carcinoma can present as a submucosal bladder tumor. CT‐guided percutaneous biopsy is a safe and effective diagnostic option in such cases. Careful pathological evaluation and individualized treatment planning are critical for accurate diagnosis and optimal management in atypical presentations of urothelial carcinoma.

## 1 Introduction

Urothelial carcinoma (UC) is the most common malignancy of the urinary tract. It typically arises from the mucosal surface of the bladder, pelvis, or ureter. Standard methods such as cystoscopy, ureterorenoscopy, urine cytology, and biopsy are generally sufficient for diagnosing mucosal lesions. In contrast, diagnosis of submucosal lesions without an apparent mucosal abnormality, which are rare, can be challenging [1]. We present an unusual case of intramural ureteral UC which arose at the ureterovesical junction and presented as a submucosal bladder mass. Diagnosis was achieved using a percutaneous trans‐extraperitoneal and transvesical computed tomography (CT)‐guided needle biopsy.

## 2 Case Presentation

**FIGURE 1:**
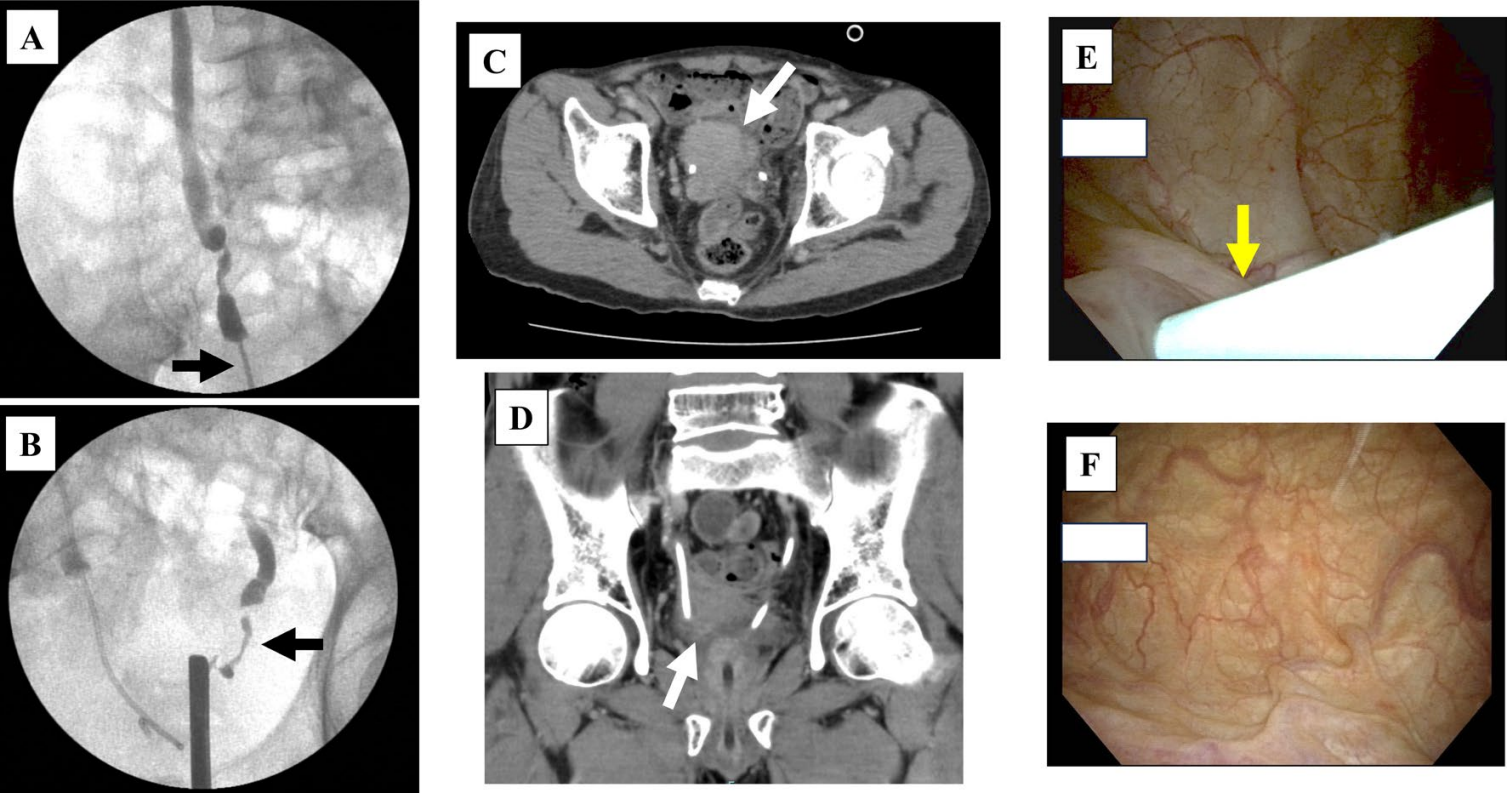
Radiographic and cystoscopic images of the bilateral ureters, submucosal bladder tumor, and bladder mucosa. Retrograde pyeloureterography (A, right ureter; B, left ureter) revealed distal ureteral strictures in both ureters (black arrows). Axial (C) and coronal (D) computed tomography images showed a 50‐mm soft‐tissue mass located at the right dorsal bladder wall (white arrow). (E, F) Cystoscopic examination revealed no mucosal abnormalities at the site of the lesion. The yellow arrow indicates the right ureteral stent.

**FIGURE 2:**
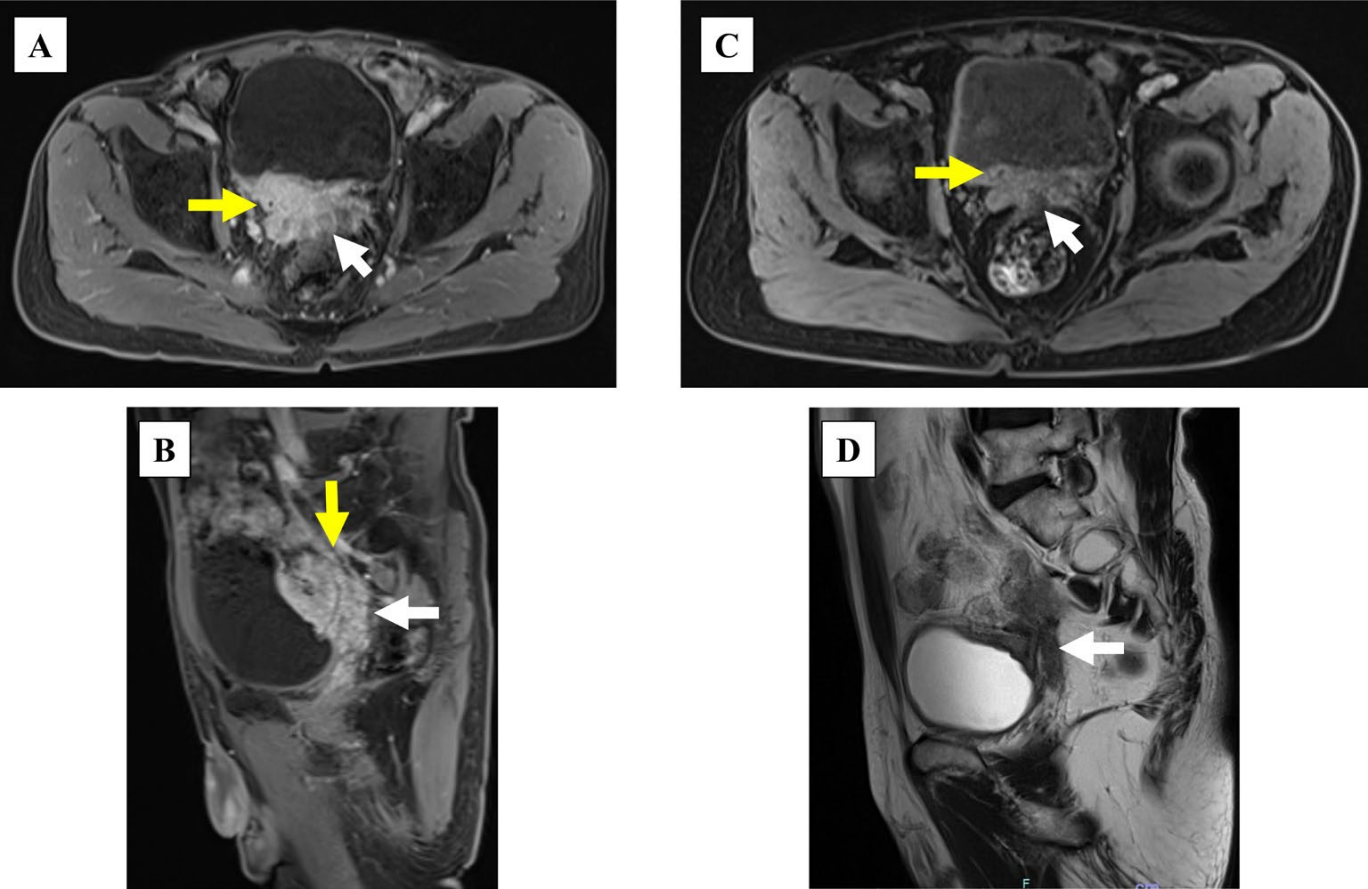
Magnetic resonance imaging of the submucosal bladder mass. Axial (A) and sagittal (B) T1‐weighted images with contrast showed a submucosal bladder mass (white arrow, 65 × 40 × 40 mm^3^). Axial T1‐weighted (C) and sagittal T2‐weighted (D) images showed shrinkage of the submucosal bladder mass (white arrow, 10 × 10 × 10 mm^3^) after two cycles of neoadjuvant chemotherapy. The yellow arrows indicate the right ureter.

A 74‐year‐old man presented with acute renal failure caused by bilateral hydronephrosis. Although retrograde pyeloureterography revealed bilateral distal ureteral strictures (Figure 1A,B), urine cytology obtained from ureteral catheters was negative for malignancy. After bilateral ureteral stents were placed and his renal function improved, contrast‐enhanced CT identified an enhancing soft‐tissue mass in the right dorsal bladder wall and right renal atrophy (Figure 1C,D and Figure S1). He was then referred to our hospital for further evaluation and treatment. His past medical history was notable for open abdominal aortic aneurysm repair; he had no history of cancer. Tumor markers (PSA and NSE) were within the reference range, but soluble IL2‐receptor (720 U/mL) was slightly elevated. Cystoscopy showed no mucosal abnormalities in the bladder (Figure 1E,F). Voided urine cytology was negative. Magnetic resonance imaging demonstrated a well‐defined solid mass without apparent mucosal involvement or lymphadenopathy, raising suspicion of a submucosal bladder malignancy, such as sarcoma (Figure 2A,B).

**FIGURE 3:**
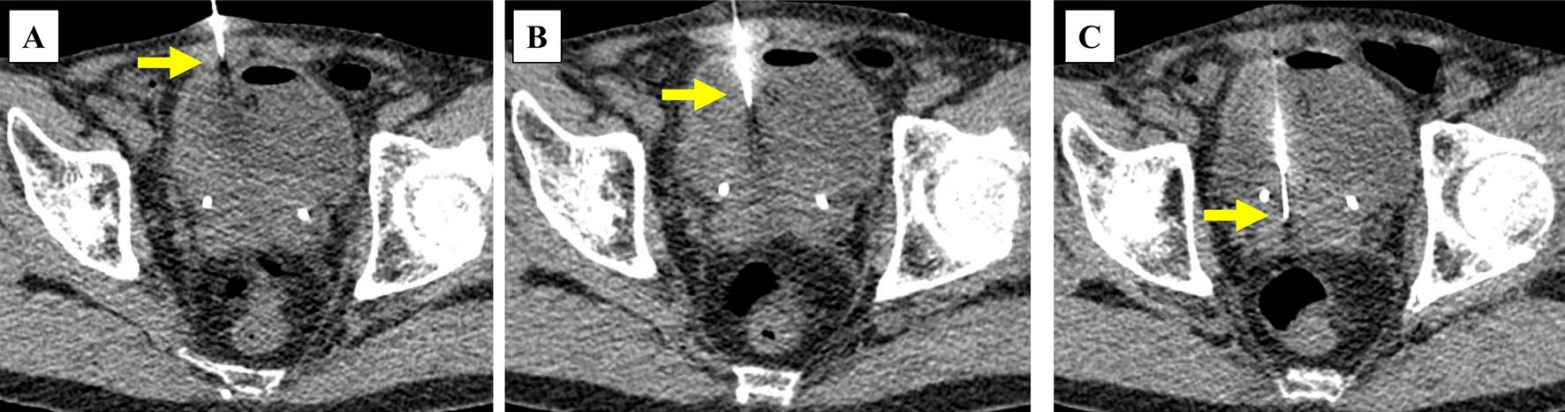
Percutaneous transvesical computed tomography‐guided needle biopsy. After distending the bladder with normal saline, an 18‐G introducer needle was placed into the bladder under image guidance (A, B). Five tissue samples from the bladder mass were obtained using a 20‐G semiautomatic biopsy needle (C). The yellow arrows indicate the position of the needle tip.

**FIGURE 4:**
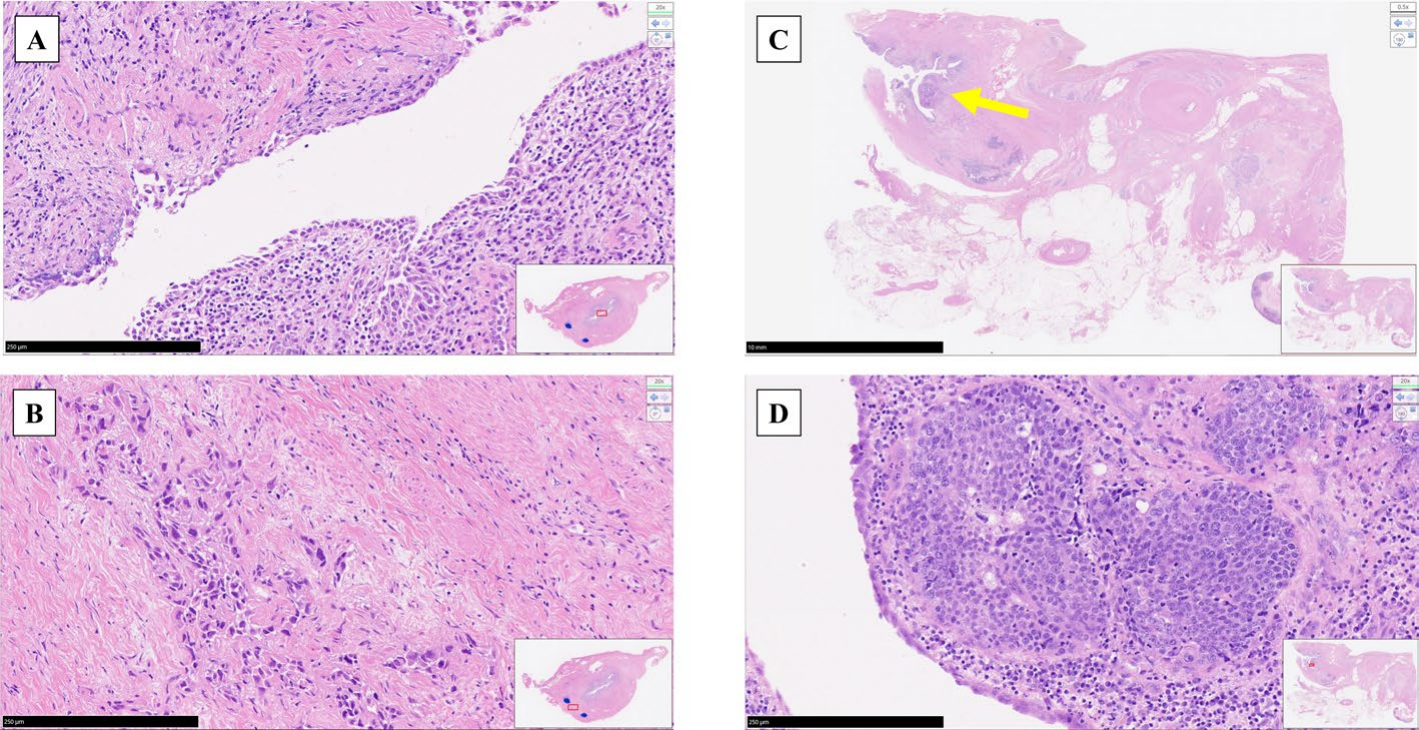
Histopathological findings of the right ureteral stump and intramural ureter. Histopathological analysis of the right ureteral stump showed normal ureteral mucosa (A) and urothelial carcinoma in the periureteral tissue (B) (×200 magnification, black bar indicates 250 μm). (C) Panoramic view of the right intramural ureter (yellow arrow) (black bar indicates 10 mm). (D) Histopathological analysis of the intramural ureter demonstrated urothelial carcinoma in the submucosal area of the right ureter (enlargement of panel C, ×200 magnification, black bar indicates 250 μm).

To obtain a definitive diagnosis while minimizing the risk of peritoneal dissemination, we performed a percutaneous trans‐extraperitoneal and transvesical CT‐guided biopsy using a coaxial needle system (Figure 3). Submucosal high‐grade UC of the bladder was diagnosed based on histopathological examination of the specimen and the imaging findings. After two cycles of neoadjuvant chemotherapy with gemcitabine and carboplatin, which resulted in tumor shrinkage (Figure 2C,D), laparoscopic radical cystectomy and ureterostomy were performed. Intraoperatively, the right ureteral stump mucosa was negative for malignancy; however, the periureteral tissue at the right ureteral stump was positive (Figure 4A,B). Additionally, extensive and severe adhesions surrounding the right ureter were observed, suggesting positive resection margin and complicating intraoperative decision‐making. Following intraoperative consultation with the patient's family regarding the risks and benefits of simultaneous right nephroureterectomy, a shared decision‐making approach led to proceeding with cystectomy alone, with close perioperative monitoring. Histopathological analysis of the cystectomy specimen showed no mucosal tumor in the bladder. However, UC lesion was primarily observed just beneath the ureteral mucosa at the ureterovesical junction (Figure 4C,D). Extensive invasion of the bladder muscle and perivesical fat was observed, and regional lymph node metastasis was identified, resulting in a final pathological diagnosis of intramural ureteral UC (ypT3N1M0) with a positive resection margin. The patient initiated treatment with immune checkpoint inhibitor 2 months after the surgery. At the 9‐month follow‐up, the patient was disease‐free with negative urine cytology from the ureterostomy, and serial CT imaging obtained every 3 months showed no metastatic disease.

## 3 Discussion

The ureterovesical junction is the anatomical interface between the ureter and the bladder. Intramural ureteral UC originates in this region and typically extends along the urothelium toward the upper ureter or bladder, eventually invading the submucosa and muscle layer. Symptoms commonly include hematuria, hydronephrosis, and frequent urination. Most cases of UC are usually detectable using ureterorenoscopy, cystoscopy, or urine cytology [2]. However, in our patient, the tumor arose from a small intramural ureteral lesion and extensively infiltrated the bladder wall. Notably, no bladder mucosal changes were apparent and no malignant cells were found in voided urine or in urine obtained from ureteral catheters. To the best of our knowledge, this is the first report of an intramural ureteral UC presenting as a submucosal bladder tumor.

Submucosal bladder UC covered by intact mucosa in a patient with no history of UC has previously been reported [1], showing that UC should be considered when evaluating bladder masses with normal overlying mucosa in patients with no prior history of UC. In that patient, a visible submucosal lesion enabled pathological diagnosis by transurethral resection. In contrast, the tumor in our patient did not visibly protrude beneath the bladder mucosa during cystoscopy. Furthermore, urine cytology from the ureteral catheters was negative, suggesting a transurethral approach would be nondiagnostic. Although brush cytology from the intramural ureter might have been diagnostic, distinguishing between dysplasia and carcinoma in situ using this method can be difficult [3]. If the patient had agreed to bilateral nephrostomy, it is possible that continued right renal pelvic urine cytology might have led to UC diagnosis.

Since submucosal bladder tumors can be benign or malignant and can arise from different cell types, histopathological confirmation is essential to guide treatment [4]. Transurethral resection (TUR) biopsy was one of the diagnostic options in this patient. However, cystoscopy revealed no clear submucosal protrusion, making TUR technically challenging. While transperitoneal biopsy was also considered, the cranial location of the mass made this approach technically difficult. Therefore, we selected CT‐guided biopsy. To avoid transperitoneal needle passage and minimize the risk of peritoneal seeding, we performed a percutaneous trans‐extraperitoneal and transvesical CT‐guided biopsy using a coaxial needle system [5, 6]. Although tumor seeding of the biopsy tract was a concern, the tract was within the surgical field for radical cystectomy; thus, any potentially contaminated tissue could be removed during the operation [7]. This approach safely provided an accurate diagnosis without disseminating the tumor.

In a retrospective study from Japan, only 80 of 3083 patients with UC who underwent surgical treatment had intramural ureteral cancer [2]. Of these, 41 underwent radical nephroureterectomy, 26 underwent transurethral resection alone, and 13 underwent radical cystectomy. This suggests that the surgical treatment was selected based on tumor location within the ureterovesical junction. Our patient was initially diagnosed with submucosal bladder UC and underwent radical cystectomy. He was scheduled to undergo simultaneous nephroureterectomy if the frozen section diagnosis of the ureteral stump was positive. During surgery, although the right ureteral stump mucosa was negative, the surrounding ureteral tissue was positive. However, because the surgical margin of the right bladder wall was judged as positive during the operation (confirmed later), we decided to omit the simultaneous radical nephroureterectomy, considering that additional resection would not sufficiently improve margin status and could increase surgical risk.

In summary, this case highlights the diagnostic and therapeutic challenges of intramural ureteral UC presenting as a submucosal bladder tumor with intact bladder mucosa and negative urine cytology. Careful histopathological evaluation and individualized surgical planning are essential to achieve accurate diagnosis and optimal patient outcomes.

## Consent

The patient provided consent for publication of this case report and any accompanying images.

## Conflicts of Interest

The authors declare no conflicts of interest.

## Supporting information


**Figure S1:** Right renal atrophy was demonstrated on an axial computed tomography image.

## Data Availability

The data that support the findings of this study are available from the corresponding author upon reasonable request.

## References

[1] F. Niimi , T. Danno , S. Iwata , S. Honda , S. Itagaki , and T. Azuma , “Submucosal Urothelial Bladder Cancer: A Case Report,” Molecular and Clinical Oncology 14, no. 1 (2021): 77, 10.3892/mco.2021.2239. 33680465 PMC7922849

[2] T. Oshina , S. Taguchi , J. Miyakawa , et al., “Clinicopathological Features and Oncological Outcomes of Urothelial Carcinoma Involving the Ureterovesical Junction,” Japanese Journal of Clinical Oncology 52, no. 1 (2022): 65–72, 10.1093/jjco/hyab143. 34510192

[3] L. G. Dodd , W. W. Johnston , C. N. Robertson , and L. J. Layfield , “Endoscopic Brush Cytology of the Upper Urinary Tract. Evaluation of Its Efficacy and Potential Limitations in Diagnosis,” Acta Cytologica 41, no. 2 (1997): 377–384, 10.1159/000332528. 9100770

[4] A. Ohtsu , S. Arai , Y. Fujizuka , et al., “Retroperitoneal Urothelial Carcinoma Arising After Bladder Diverticulectomy: A Case Report,” BMC Urology 23, no. 1 (2023): 88, 10.1186/s12894-023-01266-x. 37165362 PMC10173469

[5] T. F. Nunes , T. K. Tibana , R. F. T. Santos , B. B. de Faria , V. A. V. Fornazari , and E. Marchiori , “Percutaneous Access for the Diagnosis of Urothelial Neoplasms: Pictorial Essay With Anatomopathological Correlation,” Radiologia Brasileira 53, no. 5 (2020): 345–348, 10.1590/0100-3984.2019.0091. 33071379 PMC7545735

[6] S. R. Butros , C. J. McCarthy , A. D. Karaosmanoğlu , A. S. Shenoy‐Bhangle , and R. S. Arellano , “Feasibility and Effectiveness of Image‐Guided Percutaneous Biopsy of the Urinary Bladder,” Abdominal Imaging 40, no. 6 (2015): 1838–1842, 10.1007/s00261-015-0356-5. 25875861

[7] A. Gusev , S. Greenberg , S. Dave , A. Sobieh , and J. Yates , “Percutaneous Biopsy Tract Seeding in a Patient With Muscle‐Invasive Bladder Cancer,” European Urology Open Science 21 (2020): 17–21, 10.1016/j.euros.2020.07.005. 34337464 PMC8317869

