# Supplementary figures and images for: Computed Tomography‐Guided Biopsy of a Ureteral Urothelial Carcinoma Mimicking a Submucosal Bladder Tumor at the Ureterovesical Junction
Source: IJU Case Rep. 2025 Dec 21;9(1):e70132. doi: 10.1002/iju5.70132 (PMC12719012; doi:10.1002/iju5.70132)

## Slide 1
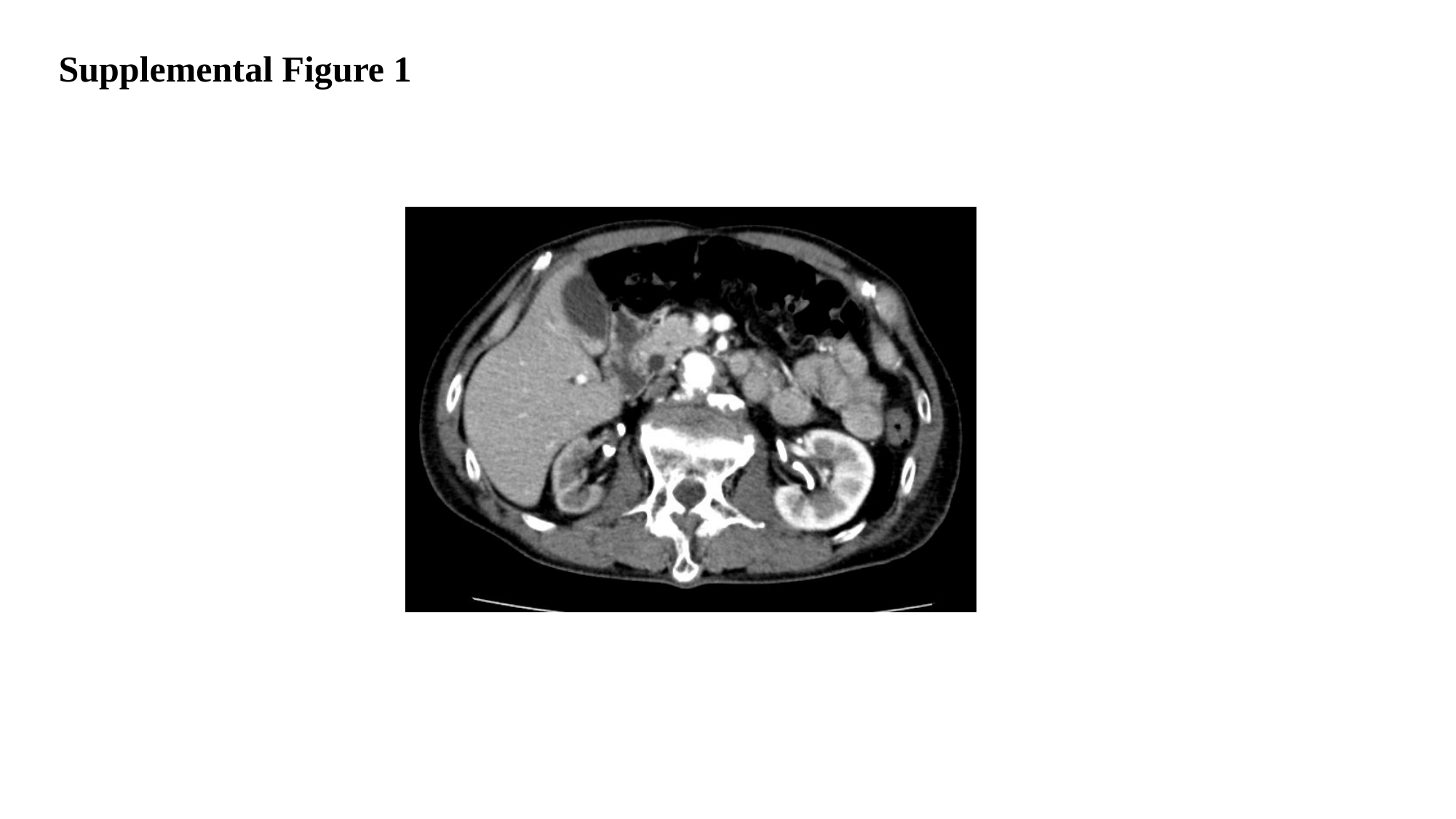

Supplemental Figure 1

Supplement: Supplementary file 1 — Figure S1: Right renal atrophy was demonstrated on an axial computed tomography image. [file IJU5-9-e70132-s001.pptx]
